# Tai Chi for Dynamic Balance Training Among Individuals with Cerebellar Ataxia: An Assessor-Blinded Randomized-Controlled Trial

**DOI:** 10.1089/jicm.2021.0222

**Published:** 2022-02-11

**Authors:** Stanley John Winser, Marco Pang, William W.N. Tsang, Susan L. Whitney

**Affiliations:** ^1^ Department of Rehabilitation Sciences, The Hong Kong Polytechnic University, Hung Hom, Hong Kong.; ^2^ Department of Physiotherapy, The Open University of Hong Kong, Hong Kong, Hong Kong.; ^3^ School of Health and Rehabilitation Sciences, University of Pittsburgh, Pittsburgh, PA, USA.

**Keywords:** cerebellar ataxia, Tai Chi, dynamic balance, postural stability

## Abstract

***Objective:*** To evaluate the immediate and long-term effects of 12 weeks of Tai Chi training on dynamic balance and disease severity among individuals with cerebellar ataxia (CA).

***Design:*** An assessor-blinded, two-arm, parallel-group randomized-controlled trial was conducted among 24 participants with CA. Participants were randomized to receive either Tai Chi intervention (*n* = 12) or usual care (*n* = 12). Dynamic balance was assessed using the Berg Balance Scale (BBS), Scale for the Assessment and Rating of Ataxia (SARA) balance sub-component of the SARA (SARAbal), Sensory Organization Test, and Limits of Stability test. Disease severity was assessed using the SARA and health-related quality of life using the EuroQol visual analog scale. Assessments were completed at baseline (week 0: T1), postintervention (week 12: T2), and at the end of the 24-week (week 36: T3) follow-up period.

***Interventions:*** The 8-form Tai Chi exercise was delivered in 60-min sessions, three times a week for 12 weeks. Participants were asked to complete an unsupervised home Tai Chi exercise program over the next 24 weeks. Participants in the usual care control group completed all study measures but did not receive any intervention.

***Results:*** Compared with the usual care control group, after 12 weeks of Tai Chi training, the experimental group demonstrated beneficial effects for dynamic balance assessed using the BBS (mean difference [MD]: 4, 95% confidence interval [CI]: −1.06 to 8.71) and the SARAbal (MD: −1.33, 95% CI: −2.66 to 2.33). The effect size ranged from small to large. The benefits gained were not sustained after 24 weeks during the follow-up assessment. Tai Chi did not benefit disease severity and health-related quality of life in this population.

***Conclusion:*** Some evidence supports the immediate beneficial effects of 12 weeks of Tai Chi training on the dynamic balance among individuals with CA.

Australia New Zealand Clinical Trials Registry (ACTRN12617000327381).

## Introduction

Cerebellar ataxia (CA) is an umbrella term that refers to genetically and nongenetically inherited conditions characterized by postural instability and the incoordination of gait, speech, limb, and eyeball movements.^1^ Damage to the cerebellum can be caused by traumatic, vascular, congenital, or metabolic events, and in certain cases, damage occurs spontaneously.^2^ The prevalence of CA differs depending on the underlying cause. Dominant hereditary CA is estimated to have a prevalence of 2.7 cases per 100,000.^3^ Among the sporadic ataxias, multiple sclerosis is a common cause, with nearly one-third to 50% of patients at risk of developing cerebellar symptoms.^4,5^ In China, among patients with autosomal dominant spinocerebellar ataxia (SCA), the prevalence of SCA-3 is higher than that of SCA-1 and SCA-2.^6^ CA is associated with a high cost, with a mean annual cost of EUR 18,776 (HKD 176,526) per patient in Spain^7^ and a mean 6-month health care cost per patient of HKD 146,832 in Hong Kong.^8^

Poor balance and walking difficulties are hallmarks of health conditions associated with CA.^9^ In addition, frequent falls are common, and nearly 93% of individuals with CA report at least one fall within 12 months,^10^ and 87% of those are frequent fallers.^8^ Most falls result in soft tissue injuries; however, 30% of falls result in bone fractures and require hospital admission.^10^ Exercises can be beneficial for improving CA symptoms.^11^ However, a review reporting the efficacy of physiotherapy and exercise for improving balance deficits is inconclusive.^12^ Studies examining the combination of intensive physiotherapy and occupational therapy have demonstrated that this approach can improve balance^13^ and reduce the fall frequency among this population.^14^ However, evidence-based guidelines for both the assessment^15,16^ and treatment of balance problems among individuals with CA remain limited.^17^ A limited number of high-quality studies have been published in this field, and the heterogeneity of the health conditions that can result in CA is also a confounding factor.^11^

Tai Chi is a Chinese martial art form that is practised for defence and health benefits. Tai Chi is thought to improve balance by facilitating an even weight distribution between the lower limbs, improve knee joint proprioception,^18^ and increasing the muscle strength of the lower limbs. Systematic reviews have reported both the functional effectiveness^19^ and cost-effectiveness^20^ of Tai Chi for improving balance among people with Parkinson's disease. Similarly, studies have reported the benefits of Tai Chi among individuals with stroke,^21^ multiple sclerosis,^22^ and spinal cord injuries.^23^ The degree of impairment observed in balance and postural control and frequency of falls among individuals with CA are comparable with those among individuals with Parkinson's disease, which are both movement disorders. Therefore, interventions that are beneficial in people with Parkinson's disease are speculated to also benefit individuals with CA.

Importantly, the conventional Frenkel's exercise for improving coordination in individuals with CA is known to improve balance and reduce falls.^24^ The principles underlying Frenkel's exercise are based on concentration, precision, and repetition of movements.^25^ The principles that underlie Tai Chi are similar to those for Frenkel's exercise.^26^ Therefore, Tai Chi training might provide improved balance and reduced disease severity among people with CA, similar to Frenkel's exercise. A previous pilot study examining 10 individuals with CA found that Tai Chi training was a potentially beneficial, feasible, and safe method for improving balance.^26^ The lack of empirical evidence supporting the effects of Tai Chi among this population justifies the need for a randomized-controlled trial. Therefore, this study aimed to compare the immediate and long-term effects of 12 weeks of Tai Chi training against a usual care control group on dynamic balance, disease severity, and health-related quality of life among individuals with CA.

## Materials and Methods

This study was an assessor-blinded, two-arm, parallel-group randomized-controlled trial. The study protocol was based on a previously completed feasibility study^26^ and was prospectively registered with the Australia New Zealand Clinical Trials Registry. Ethical approval for this study was obtained from the Human Subjects Ethics subcommittee of Hong Kong (HSEARS20170519004). Trial registration and ethics approval were completed before the recruitment of the first participant. Individuals with CA were recruited through the Hong Kong Spinocerebellar Ataxia Association (HKSCAA) newsletter. Interested volunteers who provided written informed consent were screened for eligibility. Inclusion criteria included people with a confirmed diagnosis of CA, aged 18–65 years, able to walk at least 10 m with or without walking aids, and presenting with at least one of the following cardinal ataxia symptoms: gait ataxia (unable to perform tandem walking or presents instability while tandem walking); limb ataxia (dysdiadochokinesia or dysmetria); dysarthria (scanning speech); and nystagmus. Potential participants were excluded if they had severe cognitive or visual impairments or were only able to walk with the handheld support of another individual.

Participants who provided written informed consent were randomly allocated to receive either the Tai Chi intervention (experimental group) or usual care (control group). Randomization was performed by a clinical research administrator using a simple randomization technique (the generation of a random number list) with allocation concealment. The allocation was provided to the participants in opaque sealed envelopes. Randomization and allocation were performed before baseline assessments. Block randomization was adopted to ensure equal group sizes.^27^ The clinical research administrator was an independent individual with no other involvement in the study. Eligible participants completed a screening questionnaire regarding their demographic characteristics and details of their disease. Previous medical reports were assessed to obtain information regarding the patients' diagnoses. Outcome assessments for all participants were completed at baseline (week 0: T1), immediately after the 12-week intervention (week 12: T2), and at the end of the 24-week (week 36: T3) follow-up period. All assessments were performed by a research assistant who was blinded to group allocations. The principal investigator was blinded to the group allocations during data analysis. Each group was assigned a code that was revealed only after the analysis was completed. A second research assistant was involved in contacting the participants to arrange assessment, treatment, and follow-up appointments. The authors defined an adverse event as a fall or a near fall during the institution-based or home-based Tai Chi practice.

### Intervention

The experimental group completed supervised, institution-based Tai Chi training once every 2 weeks at the Rehabilitation Research Laboratory of the Hong Kong Polytechnic University and were asked to perform unsupervised, home-based Tai Chi training 3 days each week for 12 weeks, with each session lasting 60 min. Participants were encouraged to practice home-based Tai Chi on two additional and separate days during the weeks that they participated in institution-based Tai Chi training and on three separate days during the weeks when institution-based training was not scheduled. During the follow-up period, the participants of the experimental group were instructed to continue unsupervised Tai Chi exercises at home with a similar dosage. A certified Tai Chi master with 25 years of experience delivered the supervised, institution-based Tai Chi training for the experimental group. The institution-based Tai Chi training was delivered in groups, with each group consisting of no more than five participants. Participants were trained to perform the 8-form Tai Chi routine, which is derived from Yang's style of traditional long-form Tai Chi.^28^ The 8-form Tai Chi routine can be performed either sitting or standing and has been specifically designed for individuals with difficulty standing. Performing Tai Chi in a seated position is effective for improving balance among individuals with spinal cord injury^13^ and community-dwelling older adults.^29^ Study participants who experienced difficulty standing for the entire 60-min session were offered Tai Chi moves that could be performed in a seated position or while leaning against a wall. To ensure the safety of all participants, caregivers were allowed to remain nearby the participants during the performance of the Tai Chi exercises. A video DVD illustrating the 8-form Tai Chi routine in both seated and standing positions was provided to each participant. Participants were encouraged to view the DVD when performing Tai Chi exercises at home. Participants were also provided with a booklet containing written instructions for how to perform the Tai Chi exercises and pictures of each form. An adherence diary was provided to all participants, and they were requested to record each time they completed the Tai Chi training exercises at home. Participants were asked to record any adverse events, including falls or injuries, that occurred during the practice of Tai Chi exercises either at the institution or at home. A detailed description of the instructions and forms provided to each participant has been published as an appendix elsewhere.^26^

Participants assigned to the usual care control group did not receive the trial intervention but completed all other study outcome measures. They were offered one oral session on the importance of preventing falls among individuals with CA. The session did not include any demonstrations or exercises. They were free to continue their usual activities during the trial period; however, they were not allowed to participate in any new interventions for improving balance until the end of the trial. Two complementary Tai Chi sessions of 60-min duration led by the Tai Chi instructor and Tai Chi demonstration DVD were offered to all control group participants at the end of the trial study period.

### Outcome measures

The Berg Balance Scale (BBS) is a measure of dynamic balance^30^ that includes 14 items that test balance in seated and standing positions, with each item rated between 0 and 4. This measure is scored out of 56 total points, with higher scores indicating better balance. The BBS is recommended as a standardized measure of balance for CA, with good reliability and validity.^31,32^

The Scale for the Assessment and Rating of Ataxia (SARA) is a measure used to rate ataxia severity.^33^ The SARA comprises eight subcomponents; however, scoring is not equal across the subcomponents. The SARA can have a total score of 40, with higher scores interpreted as severe ataxia symptoms. The SARA is reported to have good reliability,^33^ validity,^33^ and responsiveness.^34^

The gait, stance, and sit subcomponents of the SARA comprise the SARA balance sub-component of the SARA (SARAbal).^31^ Each subcomponent is scored according to performance, with higher scores indicating worse balance due to CA. This measure has a maximum score of 18 (8 for gait, 6 for stance, and 4 for seated balance). The SARAbal is recommended for the clinical assessment of balance in patients with CA and has good estimated reliability and validity.^31^

The Sensory Organization Test (SOT) is a laboratory-based assessment designed to test the sensory interactions involved in maintaining balance.^35^ The Bertec Balance Advantage™ Dynamic CDP System, which features a motion base with dual-balance plates and immersive virtual reality visual surround, was used to assess the SOT. The participants stood on the motion base with dual-balance plates such that both medial malleoli were aligned with the foot marking. A harness was used for all of these participants to ensure safety. The participants were instructed to look at the immersive virtual reality visual surround screen placed in front of them and to follow the instructions of the assessor.

Using the SOT, the balance was tested across six sensory conditions, which included a combination of occluded vision, visual references, and a swaying surface. The participants were instructed to stand still during each condition, each of which lasted 20 sec. The equilibrium score was obtained using the center of gravity sway for each condition, which included a somatosensory (SOT-SOM) score, a vestibular (SOT-VES) score, a visual (SOT-VIS) score, and a composite equilibrium (SOM-COMP) score. The measure demonstrates good test/retest reliability (ICC 0.68) among healthy older adults,^36^ and adequate construct validity (ρ −0.35) among individuals with unilateral and bilateral vestibular disorders.^37^

Limits of stability (LOS) is a laboratory-based assessment for dynamic balance that can be assessed using the Bertec Balance Advantage. The LOS estimates the ability of the participants to shift their weight in multiple directions without moving their feet.^38^ The participants were instructed to stand firmly on the motion base with dual-balance plates, such that both medial malleoli were aligned with the foot marking. At the onset of the test, the participants were instructed to lean as far as possible in a given direction to hit a target displayed on the virtual reality visual surround screen without moving their feet, lifting their heels, or losing their balance. The authors estimated the reaction time (LOS-RT), and the maximal excursion (LOS-MXE) in forward, backward, right, and left directions was evaluated. RT refers to the time delay between the command to move and the onset of movement. The RT is recorded in milliseconds, and faster RT indicates better balance function. The MXE is the maximum displacement of the center of pressure in a given direction. The MXE is recorded as a percentage, with a higher percentage indicating better dynamic control during standing. The LOS has acceptable test/retest reliability, predictive validity, and responsiveness for the assessment of balance among individuals with mild stroke.^39^

The EuroQol visual analog scale (EQ-VAS), a subcomponent of the EQ 5 Dimension, was used to assess the health-related quality of life.^40^ This self-rated questionnaire includes a VAS to rate the overall health status ranging between 0 and100, where 0 implies the worst health one can imagine and 100 being the best health status. This trial used the Chinese version of the EQ-VAS.^41^ The EQ-VAS has sufficient construct validity to estimate the general health-related quality of life across a broad range of populations with neurologic impairment.^42^

This study also included a cost-effectiveness estimation of the Tai Chi intervention. The findings of the cost-effectiveness of the intervention will be reported elsewhere.

### Sample size

Based on the findings of the previous pilot study,^26^ with the BBS as a primary outcome measure, assuming 90% power and 5% type I error and allowing for a 20% dropout rate, the sample size for the current study was estimated at 48 participants.

### Statistical analysis

All data analyses were conducted using Statistical Package for Social Sciences software (SPSS, version 22; IBM, Armonk, NY). The demographic and clinical data of the participants are presented as descriptive statistics, including the mean, standard deviation (SD), and percentage. Two-way repeated-measures analysis of variance (ANOVA) with Bonferroni correction was used to examine the within- and between-group differences. In this analysis, the independent variables were the study groups (experimental and control) and assessment time points (baseline vs. postintervention, baseline vs. follow-up). Missing data were replaced using the series mean imputation method,^43^ and analyses were conducted using the complete data set containing imputations for missing values. Since the current trial is underpowered (small sample size), interpretation of results was based on the effect size and mean differences, rather than statistical significance. Effect sizes (Cohen's *F*) were computed using the formula √ήp^2^/1 − ήp^2^, where ήp^2^ is the partial eta-squared value obtained in ANOVA. The effect size was interpreted as small if the value of Cohen's *F* was 0.10, moderate for 0.25, and large for 0.40.^44^ Checks were conducted before the analysis was performed to ensure that no assumptions were violated for performing a two-way repeated-measures ANOVA. *Sensitivity analysis:* to enhance the robustness of the study findings, the authors also conducted one-way analysis of covariance (ANCOVA) (with baseline as a covariate) to examine between-group differences for the effectiveness of the Tai Chi training immediately after 12 weeks and at the end of 24 weeks of follow-up. Levene's tests were carried out for each of the analysis.

## Results

**FIG. 1.**
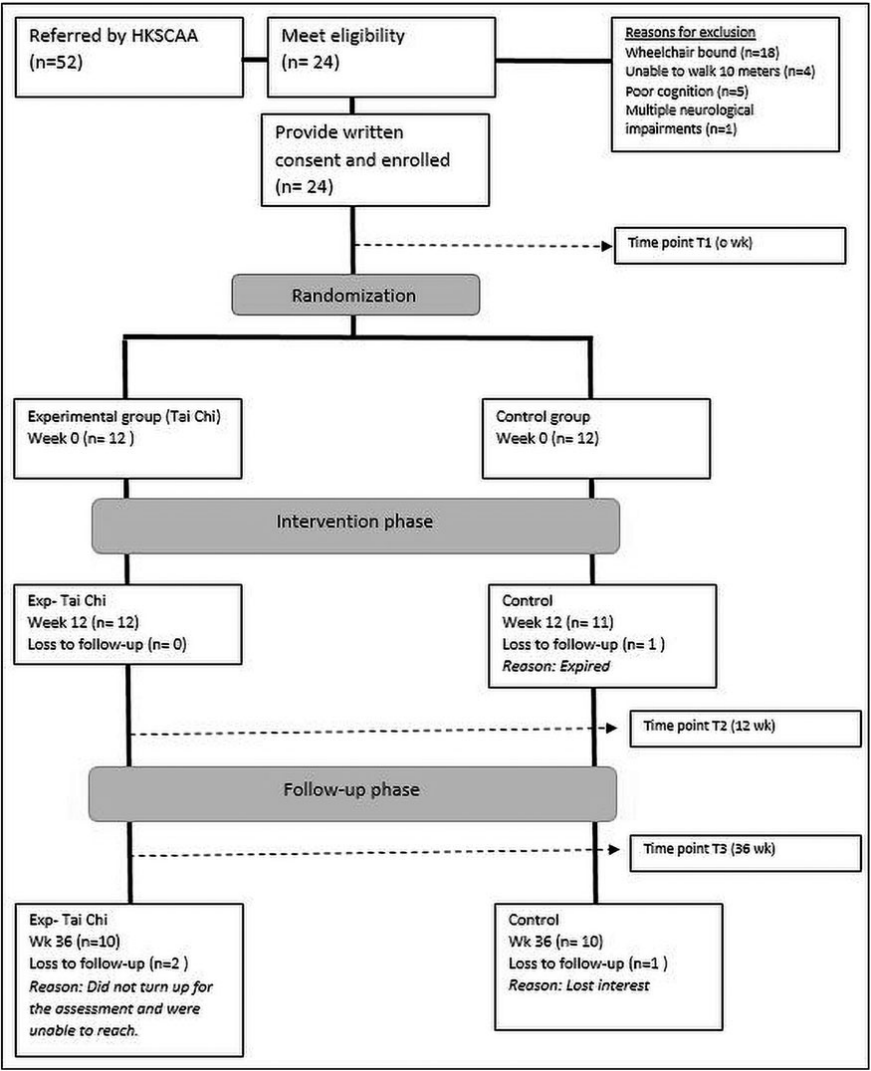
Flow of data collection.

Participants were recruited over 14 months, from December 2018 to February 2020. All included participants were recruited through the HKSCAA. Among the 52 volunteers who responded to this invitation, 24 were deemed eligible to participate. The enrolled participants were randomized into the experimental (*n* = 12) and control groups (*n* = 12). At 12 weeks, one participant from the control group was lost to follow-up, and three participants, two from the experimental and one from the control group, withdrew from the study during the 6-month follow-up assessment. The reasons for withdrawals were not related to the study intervention. Figure 1 illustrates the flow of this study. Two participants from the experimental group and three participants from the control group were unable to complete all items of the laboratory-based assessments (LOS and SOT) due to the difficulty of the test item. The missing values were replaced using the imputation method.

The baseline characteristics of the participants are presented in Table 1. There were no baseline differences in the outcome measures between the groups at T0. The mean age of the study participants was 48 years (SD: 11.3 years), and 83% of the included participants had a subtype of SCA, while the remaining were either degenerative or idiopathic ataxia. The mean attendance percentage, which is the number of attended sessions against the scheduled sessions for the institution-based workshops, was 82%, ranging between 65% and 100%. For the home-based exercises, 71% was recorded, which fell to 58% during the follow-up phase. Among the experimental group, 33% (*n* = 4) of the participants opted to lean against the wall during Tai Chi practice. Appendix Table A1 illustrates the mean scores of all outcome measures during the three assessment time lines. During the Tai Chi practice sessions, seven participants (58%) reported mild muscle pain following the first two sessions. The pain subsided completely within the 1-week period. Besides, no adverse events (falls or near falls) were recorded during the institution- or home-based exercise sessions.

**Table 1.** Demographic Data of the Included Participants (*n* = 24)

	Experimental (*n* = 12)	Control (*n* = 12)
Age, mean (SD)	48.67 (11.30)	46.89 (12.56)
Gender, *n* (%)		
Male	7 (63)	6 (50)
Female	5 (37)	6 (50)
Ethnicity, *n* (%)		
Chinese	24 (100)	24 (100)
Occupation, *n* (%)		
Employed	1 (12)	2 (17)
Unemployed	11 (75.0)	10 (83)
Retired	3 (13)	0
Diagnosis, *n* (%)		
Spinocerebellar ataxia		
SCA-1	2 (21)	2 (17)
SCA-2	0 (4)	1 (8)
SCA-3	5 (33)	4 (33)
SCA-6	2 (17)	1 (8)
SCA-11	1 (4)	0 (0)
SCA unknown type	0 (4)	0 (0)
Cerebellar degeneration	2 (17)	2 (17)
Idiopathic ataxia	0	2 (17)
Age at disease onset, mean (SD)	37.79 (10.98)	32.78 (11.22)
Disease duration, years, mean (SD)	12.71 (10.68)	15.22 (12.22)
Screening for cerebellar ataxia, *n* (%)		
Gait ataxia	12 (100)	12 (100)
Limb ataxia	10 (82)	9 (75)
Dysarthria	7 (92)	9 (75)
Nystagmus	11 (71)	12 (100)
Use of assistive walking device, *n* (%)		
Yes	9 (75)	8 (67)
No	3 (12.5)	4 (33)

SCA, spinocerebellar ataxia; SD, standard deviation.

### Tai Chi on dynamic balance

Table 2 reports the findings regarding the benefits of Tai Chi on dynamic balance assessed using the clinic-based assessments (BBS and SARAbal) and the laboratory-based assessments (LOS and SOT), disease severity using the SARA, and health-related quality of life using the EQ-VAS. A mean reduction of 1.3 points was found for the SARAbal among the experimental group, indicating balance improvement. Similarly, the mean BBS score increased by 4 points among the experimental group at the end of the intervention period (12 weeks). Effect sizes of the change in score within and between groups ranged from small to moderate for the BBS and moderate to strong for the SARAbal at 12 weeks. There were no additional benefits of the intervention compared with the control at the end of the 24-week follow-up period. Among the laboratory-based measures, SOT-SOM scores showed a mean increase of 6% among the experimental group with a large effect size. Likewise, the LOS-RT and LOS-MXE in the forward (LOS-F-RT and LOS-F-MXE) and backward directions (LOS-B-RT and LOS-B-MXE) showed improvements among the experimental groups compared with the control groups after 12 weeks of intervention. The effect size ranged from moderate to large. Except for the LOS-F-MXE, the benefits gained were not sustained after 24 weeks during the follow-up assessment. The sensitivity analysis reported a significant between-group difference for the BBS, SARAbal, SOT-SOM, and SOT-VIS at the end of the intervention period (12 weeks) and SOT-VES and LOS-F-MXE alone at the end of the 24 weeks of follow-up period. Appendix Table A2 reports the summary table of the sensitivity analysis.

**Table 2.** Mean and Standard Deviation of the Outcome Measures at Pretest, Post-Test, and 6 Months

Outcome measures	Effect of time	Between-group effect	Mean difference
	F-value; partial eta squared, effect size (*p*)		Mean (SD)[95% CI]
Clinic-based measures of balance			
BBS			
Pre vs. post	0.22; 0.01, 0.10 (0.04)^*^	0.82; 0.04, 0.38 (0.03)^*^	Exp: 3.83 (7.7) [−1.06 to 8.71] Con: −2.67 (3.7) [−5.01 to −0.33]
Pre vs. follow-up	0.35; 0.02, 0.14 (0.56)	0.37; 0.02, 0.14 (0.55)	Exp: 1.83 (4.6) [−1.11 to 4.77] Con: −0.75 (4.4) [−3.54 to 2.04]
SARAbal			
Pre vs. post	4.25; 0.16, 0.44 (0.05)^*^	0.63; 0.03, 0.18 (0.04)^*^	Exp: −1.33 (3.1) [−2.66 to 2.33] Con: 0.75 (1.5) [−0.23 to 1.73]
Pre vs. follow-up	4.46; 0.17, 0.45 (0.05)^*^	1.39; 0.06, 0.25 (0.25)	Exp: 0.33 (0.89) [−0.23 to 0.90] Con: 1.00 (2.0) [−0.27 to 2.27]
Laboratory-based measures of balance			
SOT-SOM			
Pre vs. post	2.6; 0.11, 0.35 (0.12)	28.5; 0.56, 1.13 (<0.01)^*^	Exp: 6.00 (7.1) [1.48 to 10.53] Con: −0.40 (9.7) [−6.60 to 5.77]
Pre vs. follow-up	0.72; 0.03, 0.18 (0.41)	28.8; 0.57, 1.15 (<0.01)^*^	Exp: 4.83 (5.6) [1.27 to 8.39] Con: −2.28 (8.8) [−7.89 to 3.33]
SOT-VIS			
Pre vs. post	1.43; 0.06, 0.25 (0.25)	0.64; 0.03, 0.28 (0.43)	Exp: −11.13 (15.8) [−21.18 to −1.08] Con: 18.50 (14.4) [9.38 to 27.62]
Pre vs. follow-up	0.009; 0.0001, 0.01 (0.93)	2.1; 0.09, 0.31 (0.16)	Exp: −11.68 (15.9) [−21.78 to −1.60] Con: 11.00 (19.7) [−1.50 to 23.50]
SOT-VES			
Pre vs. post	1.43; 0.06, 0.25 (0.25)	0.64; 0.03, 0.18 (0.43)	Exp: −2.30 (22.86) [−16.82 to 12.22] Con: 12.33 (14.04) [3.41 to 21.25]
Pre vs. follow-up	0.009; 0.0001, 0.01 (0.93)	2.1; 0.09, 0.31 (0.16)	Exp: −10.50 (13.58) [−19.13 to −1.87] Con: 12.50 (11.19) [5.39 to 19.61]
SOT-COMP			
Pre vs. post	14.4; 0.40, 0.82 (0.001)^*^	2.65; 0.11, 0.35 (0.12)	Exp: 1.46 (10.1) [−4.97 to 7.89] Con: −0.10 (8.6) [7.64 to 18.52]
Pre vs. follow-up	7.90; 0.26, 0.59 (0.01)^*^	3.4; 0.13, 0.39 (0.08)	Exp: −0.80 (6.8) [−5.09 to 3.49] Con: 8.63 (6.9) [4.24 to 13.03]
LOS-F-RT			
Pre vs. post	7.6; 0.26, 0.59 (0.01)^*^	21.9; 0.50, 1 (0.07)	Exp: 0.01 (0.8) [−0.53 to 0.55] Con: −0.90 (0.7) [−1.33 to −0.44]
Pre vs. follow-up	7.2; 0.25, 0.58 (0.01)^*^	27.2; 0.5, 1 (0.06)	Exp: 0.08 (0.3) [−0.10 to 0.25] Con: −0.73 (0.8) [−1.24 to −0.23]
LOS-F-MXE			
Pre vs. post	16.1; 0.42, 0.85 (0.001)^*^	4.4; 0.17, 0.45 (0.05)^*^	Exp: 30.13 (25.0) [14.26 to 46.01] Con: 3.92 (15.5) [−5.95 to 13.79]
Pre vs. follow-up	18.5; 0.46, 0.92 (0.000)^*^	4.6; 0.17, 0.45 (0.04)^*^	Exp: 22.12 (17.8) [10.80 to 33.44] Con: 0.62 (4.3) [−2.10 to 3.34]
LOS-B-RT			
Pre vs. post	0.58; 0.03, 0.18 (0.45)	12.1; 0.36, 0.75 (<0.01)^*^	Exp: −0.20 (0.9) [−0.75 to 0.35] Con: 0.41 (0.4) [0.15 to 0.67]
Pre vs. follow-up	0.30; 0.01, 0.10 (0.59)	15.5; 0.41, 0.83 (0.06)	Exp: −0.33 (0.5) [−0.64 to −0.01] Con: 0.23 (0.4) [−0.03 to 0.48]
LOS-B-MXE			
Pre vs. post	1.74; 0.07, 0.27 (0.20)	1.60; 0.07, 0.27 (0.22)	Exp: 5.00 (22.5) [−9.31 to 19.32] Con: −15.54 (16) [−25.70 to −5.38]
Pre vs. follow-up	0.91; 0.04, 0.20 (0.35)	4.7; 0.18, 0.47 (0.05)	Exp: −0.06 (13) [−8.31 to 8.19] Con: −4.80 (11.9) [−12.31 to 2.77]
LOS-R-RT			
Pre vs. post	2.2; 0.56, 1.13 (0.09)	0.59; 0.03, 0.18 (0.45)	Exp: −0.40 (0.60) [−0.76 to −0.04] Con: −0.64 (0.40) [−0.87 to −0.41]
Pre vs. follow-up	1.80; 0.45, 0.90 (0.09)	0.35; 0.02, 0.14 (0.60)	Exp: −0.10 (0.2) [−0.19 to 0.07] Con: −0.30 (0.2) [−0.42 to −0.16]
LOS-R-MXE			
Pre vs. post	14.6; 0.40, 0.82 (<0.01)^*^	0.09; 0.004 (0.77)	Exp: 19.48 (23.8) [4.33 to 34.62] Con: 8.60 (9.0) [2.89 to 14.32]
Pre vs. follow-up	0.003; 0.0001, 0.01 (0.96)	0.19; 0.009, 0.10 (0.67)	Exp: 1.70 (12.2) [−6.09 to 9.42] Con: −1.40 (6.8) [−5.76 to 2.88]
LOS-L-RT			
Pre vs. post	1.15; 0.34, 0.72 (0.08)	5.2; 0.19, 0.48 (0.03)^*^	Exp: 0.35 (0.6) [−0.03 to 0.72] Con: 0.38 (0.4) [0.10 to 0.66]
Pre vs. follow-up	8.6; 0.28, 0.62 (0.07)	1.4; 0.06, 0.25 (0.25)	Exp: 0.24 (0.9) [−0.30 to 0.78] Con: 0.61 (0.5) [0.28 to 0.94]
LOS-L-MXE			
Pre vs. post	11.3; 0.34, 0.72 (<0.01)^*^	1.2; 0.05, 0.23 (0.29)	Exp: 14.8 (20.9) [1.57 to 28.10] Con: 7.6 (9.8) [1.33 to 13.82]
Pre vs. follow-up	2.1; 0.09, 0.31 (0.17)	3.9; 0.15, 0.42 (0.06)	Exp: −5.70 (8.7) [−11.18 to −0.14] Con: 1.40 (5.7) [−2.28 to 5.00]
Severity of disease			
SARA			
Pre vs. post	3.76; 0.15 (0.07)	0.78; 0.03 (0.39)	Exp: −2.21 (2.8) [−4.01 to −0.40] Con: −0.04 (2.8) [−1.85 to 1.77]
Pre vs. follow-up	0.05; 0.02 (0.49)	0.66; 0.03 (0.43)	Exp: −0.54 (1.8) [−1.70 to 0.62] Con: 1.08 (2.0) [−0.14 to 2.31]
Health-related quality of life			
EQ-VAS			
Pre vs. post	1.0; 0.04, 0.20 (0.33)	0.69; 0.03, 0.18 (0.42)	Exp: −2.02 (14.1) [−10.97 to 6.94] Con: 7.27 (11.7) [−0.19 to 41.74]
Pre vs. follow-up	0.44; 0.02, 0.14 (0.52)	1.1; 0.05, 0.23 (0.31)	Exp: −2.02 (10.6) [−8.77 to 4.74] Con: 6.02 (18.1) [−5.46 to 17.51]

^*^ indicates statistical significance (*p* < 0.05).

B, back; BBS, Berg Balance Scale; COMP, composite; EQ-VAS, EuroQol visual analog scale; F, front; L, left; LOS, limits of stability; MXE, maximal excursion; R, right; RT, reaction time; SARA, Scale for the Assessment and Rating of Ataxia; SARAbal, balance subcomponent of the SARA; SD, standard deviation; SOM, somatosensory; VES, vestibular; VIS, visual.

### Tai Chi on disease severity

The mean disease severity score assessed using the SARA was reduced by 2 points among the experimental group at the end of the 12-week intervention, indicating an improvement in the disease severity; however, the difference between the two groups was suggestive of no additional beneficial effect for the intervention compared with the control (Appendix Table A2).

### Tai Chi on health-related quality of life

ANOVA demonstrated no beneficial effect of the intervention on the health-related quality of life assessed using the EQ-VAS at the end of 12 weeks or during the follow-up assessment after 24 weeks.

## Discussion

This study aimed to explore the benefits of Tai Chi intervention on dynamic balance, disease severity, and health-related quality of life among individuals with CA. Immediate beneficial effects were observed following the Tai Chi intervention on balance, as assessed using the BBS, SARAbal, SOT-SOM, LOS-F-RT, LOS-B-RT, LOS-F-MXE, and LOS-B-MXE. The long-term benefits of Tai Chi were limited as the benefits attained at the end of 12 weeks were not sustained during the follow-up assessment after 24 weeks. Tai Chi exercises did not have additional beneficial effects compared with control on disease severity and health-related quality of life in this population.

Impaired balance among patients with CA is associated with poor rhythmic muscle contractions,^45^ deficits secondary to movement planning and initiation,^46^ increased postural sway,^4^ and impairments in the anticipatory postural response.^10^ Exercise-based rehabilitation interventions designed to improve balance among this population typically target one or more of these deficits. Tai Chi involves moving the whole body in slow, smooth, and diagonal patterns that involve multiple joints.^47^ The movement patterns are typically repeated until they are mastered. In addition, the base of support is constantly altered for every posture to facilitate an even weight distribution, and a stable stance is emphasized during practice. First, an increase in dynamic balance likely occurred in response to the persistent weight shifts between the lower limbs that occur during Tai Chi practice, which challenges the base of support, similar to the useful changes in dynamic balance observed among individuals with Parkinson's disease.^48^ Second, the repeated practice of diagonal patterns involving the trunk and upper and lower limbs is likely to improve coordination and rhythmic muscle contractions between agonists and antagonists, improving overall stability and coordination. Third, improved SOT-SOM scores indicate improved proprioceptive inputs from the trunk and extremities, which can influence balance. These findings are in line with previous literature describing the benefits of Tai Chi on balance in people with stroke.^49^

The intervention did not have additional benefits on the disease severity compared with the control. The SARA estimates disease severity by quantifying the incoordination of gait, standing, sitting, limbs, and speech. The combination of gait, standing, and sitting comprises the SARAbal, which improved with Tai Chi practice. Except for speech, improvements in all other limb coordination-related domains were observed with repeated Tai Chi practice. However, comparing with the control group, the experimental group did not have any additional benefits. Treatment dosage could account for the lack of difference between the groups. Three sessions per week with supervised sessions only once every two weeks for 12 weeks might have been insufficient to demonstrate a difference between the groups. Future studies are warranted to increase the treatment dosage from 12 weeks to either 18 or 24 weeks to re-examine the significance of Tai Chi on disease severity among people with CA.

This feasibility study recruited 10 participants with CA within 2 weeks.^26^ However, for the present study, the recruitment period was over 12 months and despite the long recruitment period, the authors were unable to reach the desired sample size of 48. The challenge in recruiting sufficient participants was due to the stringent eligibility criteria set for this study. From the feasibility study, they found that 40% of the included participants were unable to complete the LOS and SOT as the tests were too challenging. Therefore, to address this issue and to include participants with reasonable balance and walking ability, they included participants who can walk at least 10 m with or without a walking assistive device. In this study, all the included participants underwent the SOT and LOS, and only five participants (20%) were unable to complete all the tasks. Although the response to the SOT and LOS increased, this strategy compromised the number of participants included. Future studies in this region need to consider recruiting participants from the public hospitals of Hong Kong, Mainland, and other neighboring countries for increasing the sample size.

Adherence to institution-based Tai Chi training was 82%, and adherence was lower for home-based exercises. Adherence of less than 60% during the follow-up period could be accounted for the loss of retention of the attained benefits in balance soon after the intervention phase. Second, most individuals rely on public transportation. Although the transportation system in Hong Kong is disability-friendly, 60% of these participants hired a taxi or took the rehabilitation bus organized by the HKSCAA to commute to the institution for Tai Chi sessions. To account for the unavailability of the rehabilitation bus and the financial constraints associated with hiring a taxi for each visit, this study organized Tai Chi master-led workshops only once every 2 weeks. Participants may not have completely understood the Tai Chi moves, which may have reduced their involvement in the home-based exercises. Increasing the number of directly supervised Tai Chi workshop sessions is likely to increase the treatment benefits. Therefore, future studies are recommended that conduct experiments including one or more directly supervised sessions each week, with no less than three sessions per week for 12 weeks or longer to provide accurate estimates of treatment effects.

### Strengths of the study

This study is important because it is the first of its kind. This study has several strengths. (1) Stringent methodological procedures were adopted to ensure the quality of this study. (2) A standardized set of measures were used for the assessment of dynamic balance, disease severity, and health-related quality of life to ensure the accuracy of the study findings. (3) Four outcome measures were used to assess dynamic balance, with a combination of clinic-based and laboratory-based assessments, to improve the accuracy of the findings. (4) Appropriate measures were taken to ensure adequate home-based practice, including the distribution of a home-practice DVD and adherence diary to track the involvement of the participants in the intervention.

### Study limitations

This study has the following limitations. (1) The study aimed to recruit 48 participants, however, due to the stringent inclusion criteria and the invitation of only those patients who are ambulant, only 24 participants were successfully recruited, resulting in 12 participants for each group suggestive of an underpowered trial. Considering the lack of power due to the small sample size, the findings of this study have been discussed focusing on effect size estimates and group differences rather than testing hypotheses. Therefore, the finding of this study has to be interpreted with caution. A *post hoc* power analysis for the recruited samples (*n* = 24) revealed a power of 72%. Hong Kong is a small city, with ∼250 patients with CA; this sample of 24 represents 10% of the total population, which may be considered a representative sample. However, a future study is recommended using a sample size of at least 48 subjects to explore the significance of using Tai Chi for improving balance, disease severity, and health-related quality of life in this population. (2) The findings of this study are limited to individuals with genetic and sporadic ataxia who are ambulant. This sample did not include any participants with an acquired cause, such as cerebellar stroke. The disease progression and symptoms differ among patients with acquired causes of ataxia, and balance impairments are more common among nonambulant and less ambulant patients than among ambulant patients. Future studies are recommended that recruit patients with acquired lesions and those who are nonambulant or less ambulant to increase the generalizability of the study findings. (3) This reporting on adverse events must be interpreted with caution as the authors considered falls and near falls alone as adverse events. Although this study reports no adverse events, it is acknowledged that Tai Chi intervention results in mild muscle pain that subsides within 2 weeks among at least 25% of patients with CA. Adequate warm-up exercises before Tai Chi may help to prevent muscle pain. (4) Last, although this study blinded the assessor and statistical analysis, due to the nature of the study design they were not able to blind the participants. Therefore, the risk of bias due to the lack of blinding cannot be eliminated. Lack of participant blinding may have exaggerated the estimated effect size,^50^ likewise, they are unable to comment on the placebo effects of the intervention. Future studies are recommended to address this limitation on participant blinding while designing the methodology.

## Conclusion

This study demonstrates some evidence to support the benefits of Tai Chi training on the dynamic balance among ambulant patients with CA. The effect size of treatment ranged from small to large. The changes evident after 12 weeks of intervention were not sustained at 24 weeks. Traditional Tai Chi moves and modified versions that can be performed while seated or leaning against a wall were found to be safe and appropriate for use in patients with CA. Tai Chi did not improve the severity of the disease and health-related quality of life among this population. A future study with a sample size of at least 48 participants remains necessary to consolidate these findings. Less ambulant individuals and those with acquired causes for CA need to be tested to increase the generalizability of the study findings.

## Supplementary Material

Supplemental data

## Appendix

**Appendix Table A1.** Mean and Standard Deviation of the Outcome Measures at Pretest, Post-Test, and 6 Months

Clinic-based outcome measures of balance, mean (SD)						
Outcome measure (maximum score)	Pretest (week 0: T1)		Post-test (week 12: T2)		Follow-up (week 36: T3)	
	Exp	Con	Exp	Con	Exp	Con
BBS (56)	28.08 (15.77)	25.00 (20.13)	31.91 (11.92)	22.33 (20.29)	29.92 (15.92)	24.25 (19.07)
SARAbal (18)	9.58 (3.63)	10.50 (3.99)	8.25 (3.28)	9.75 (4.67)	8.58 (3.29)	10.75 (4.12)
*Laboratory-based measures of balance*						
SOT						
SOM (100)	88.67 (4.63)	102.5 (8.54)	94.67 (5.95)	102.1 (6.17)	93.5 (4.65)	100.22 (5.17)
VIS (100)	80.13 (13.62)	61.33 (14.55)	69.00 (18.05)	79.83 (9.87)	68.44 (17.24)	72.33 (16.31)
VES (100)	80.5 (14.25)	51.5 (12.45)	78.2 (19.54)	63.83 (9.86)	70.00 (18.67)	64.00 (13.24)
COMP (100)	67.69 (7.05)	56.87 (10.62)	73.39 (8.09)	56.77 (10.62)	69.15 (10.88)	69.95 (5.85)
LOS						
RT-F (ms)	0.79 (0.62)	2.20 (0.55)	0.80 (0.6)	1.32 (0.76)	0.87 (0.57)	1.47 (0.49)
MXE-F (100)	40.92 (19.26)	69.22 (22.16)	71.05 (25.58)	73.14 (13.7)	63.04 (20.58)	69.84 (21.87)
RT-R (ms)	1.54 (0.63)	1.54 (0.13)	1.14 (0.45)	0.90 (0.44)	1.47 (0.66)	1.25 (0.2)
MXE-R (100)	86.78 (19.99)	90.99 (6.84)	106.25 (14.86)	99.59 (7.64)	88.44 (20.57)	89.55 (11.08)
RT-B (ms)	1.88 (0.79)	0.80 (0.26)	1.68 (0.88)	1.21 (0.43)	1.55 (0.66)	1.03 (0.26)
MXE-B (100)	84.43 (28.21)	103.92 (15.31)	89.43 (18.64)	88.38 (17.0)	84.37 (22.61)	99.15 (10.01)
RT-L (ms)	1.14 (0.74)	0.71 (0.27)	1.49 (0.62)	1.09 (0.26)	1.38 (0.78)	1.32 (0.56)
MXE-L (100)	89.02 (20.66)	96.94 (6.99)	103.85 (6.81)	104.52 (10.61)	83.36 (18.17)	98.30 (6.76)
*Severity of disease*						
SARA (40)	18.17 (5.22)	19.50 (7.87)	15.96 (6.72)	19.46 (7.29)	17.63 (4.78)	20.58 (7.72)
*Health-related quality of life*						
EQ-VAS (100)	81.18 (10.8)	72.73 (13.88)	79.17 (16.07)	80.00 (10.23)	79.17 (12.58)	78.75 (14.0)

B, back; BBS, Berg Balance Scale; COMP, composite; EQ-VAS, EuroQol visual analog scale; F, front; L, left; LOS, limits of stability; MXE, maximal excursion; R, right; RT, reaction time; SARA, Scale for the Assessment and Rating of Ataxia; SARAbal, balance sub-component of the SARA; SD, standard deviation; SOM, somatosensory; VES, vestibular; VIS, visual.

**Appendix Table A2.** Findings of the Sensitivity Analysis Reporting the Mean Between-Group Differences at Postintervention and 6 Months Using Analysis of Covariance

Outcome measures	Between-group effect	Mean between-group difference
	F-value; partial eta squared (*p*)	Mean (standard error) [95% CI]
Clinic-based measures of balance		
BBS		
Postintervention	8.85; 0.30 (0.05)^*^	Exp vs. Con: 6.9 (2.32) [2.08 to 11.73]
Follow-up	2.26; 0.10 (0.15)	Exp vs. Con: 2.77 (1.84) [−1.06 to 6.59]
SARAbal		
Postintervention	0.05; 0.002 (0.04)^*^	Exp vs. Con: −2.22 (1.00) [−1.79 to 2.24]
Follow-up	2.14; 0.09 (0.16)	Exp vs. Con: −0.89 (0.61) [−2.15 to 3.75]
Laboratory-based measures of balance		
SOT-SOM		
Postintervention	2.5; 0.11 (0.01)^*^	Exp vs. Con: 5.76 (3.64) [−1.81 to 13.33]
Follow-up	2.2; 0.10 (0.15)	Exp vs. Con: 4.28 (2.89) [−1.72 to 10.29]
SOT-VIS		
Postintervention	8.59; 0.29 (0.008)^*^	Exp vs. Con: −19.45 (6.64) [−33.25 to −5.65]
Follow-up	2.04; 0.09 (0.17)	Exp vs. Con: −11.49 (8.04) [−28.20 to 5.23]
SOT-VES		
Postintervention	0.99; 0.46 (0.33)	Exp vs. Con: 9.61 (9.67) [−10.50 to 29.72]
Follow-up	5.01; 0.19 (0.04)^*^	Exp vs. Con: −17.17 (7.67) [−33.11 to −1.22]
SOT-COMP		
Postintervention	1.98; 0.09 (0.17)	Exp vs. Con: −5.44 (3.86) [−13.47 to 2.60]
Follow-up	3.79; 0.15 (0.07)	Exp vs. Con: −5.93 (3.05) [−12.27 to 0.41]
LOS-F-RT		
Postintervention	0.04; 0.002 (0.84)	Exp vs. Con: −0.09 (0.44) [−1.01 to 0.83]
Follow-up	0.03; 0.01 (0.88)	Exp vs. Con: −0.05 (0.32) [−0.72 to 0.62]
LOS-F-MXE		
Postintervention	1.62; 0.07 (0.22)	Exp vs. Con: −11.67 (9.16) [−30.73 to 7.38]
Follow-up	6.78; 0.24 (0.02)^*^	Exp vs. Con: −16.63 (6.39) [−29.92 to 3.35]
LOS-B-RT		
Postintervention	0.07; 0.003 (0.79)	Exp vs. Con: −1.10 (0.36) [−0.84 to 0.65]
Follow-up	0.19; 0.009 (0.67)	Exp vs. Con: −0.09 (0.22) [−0.54 to 0.36]
LOS-B-MXE		
Postintervention	2.01; 0.09 (0.17)	Exp vs. Con: 9.60 (6.78) [−4.49 to 23.70]
Follow-up	0.25; 0.01 (0.62)	Exp vs. Con: −2.18 (4.35) [−11.23 to 6.87]
LOS-R-RT		
Postintervention	2.08; 0.09 (0.16)	Exp vs. Con: 0.24 (0.16) [−0.11 to 0.59]
Follow-up	7.04; 0.25 (0.02)^*^	Exp vs. Con: 0.23 (0.09) [0.05 to 0.41]
LOS-R-MXE		
Postintervention	2.00; 0.09 (0.17)	Exp vs. Con: 7.02 (4.96) [−3.29 to 17.34]
Follow-up	0.41; 0.02 (0.41)	Exp vs. Con: 2.65 (4.12) [−5.91 to 11.21]
LOS-L-RT		
Postintervention	1.36; 0.06 (0.26)	Exp vs. Con: 2.17 (0.17) [−0.17 to 0.60]
Follow-up	0.20; 0.01 (0.66)	Exp vs. Con: −0.29 (0.29) [−0.72 to 0.47]
LOS-L-MXE		
Postintervention	0.002; 0.00 (0.96)	Exp vs. Con: 0.19 (3.79) [−7.70 to 8.07]
Follow-up	9.58; 0.31 (0.01)^*^	Exp vs. Con: −8.80 (2.83) [−14.64 to −2.87]
Severity of disease		
SARA		
Postintervention	3.50; 0.14 (0.06)	Exp vs. Con: −2.22 (1.19) [−4.69 to −0.25]
Follow-up	5.17; 0.20 (0.07)	Exp vs. Con: −1.73 (0.76) [−3.31 to −0.15]
Health-related quality of life		
EQ-VAS		
Postintervention	1.10; 0.05 (0.31)	Exp vs. Con: −5.42 (5.17) [−16.17 to 5.34]
Follow-up	0.23; 0.01 (0.64)	Exp vs. Con: −2.65 (5.55) [−14.20 to 8.90]

^*^ indicates statistical significance (*p* < 0.05).

B, back; BBS, Berg Balance Scale; CI, confidence interval; COMP, composite; EQ-VAS, EuroQol visual analog scale; F, front; L, left; MXE, maximal excursion; R, right; RT, reaction time; LOS, limits of stability; SARA, Scale for the Assessment and Rating of Ataxia; SARAbal, balance subcomponent of the SARA; SOM, somatosensory; VES, vestibular; VIS, visual.
